# Draft genomes assembly and annotation of *Carex parvula* and *Carex kokanica* reveals stress-specific genes

**DOI:** 10.1038/s41598-022-08783-z

**Published:** 2022-03-23

**Authors:** Guangpeng Qu, Yuhong Bao, Yangci Liao, Can Liu, Hailing Zi, Magaweng Bai, Yunfei Liu, Dengqunpei Tu, Li Wang, Shaofeng Chen, Gang Zhou, Muyou Can

**Affiliations:** 1 State Key Laboratory of Hulless Barley and Yak Germplasm Resources and Genetic Improvement, Lhasa, 850000 China; 2 Institute of Grassland Science, Tibet Academy of Agriculture and Animal Husbandry Science, Lhasa, 850000 China; 3 Novogene Bioinformatics Institute, Beijing, China; 4 Tibet Academy of Agriculture and Animal Husbandry Sciences, Lhasa, China

**Keywords:** Plant stress responses, Plant sciences, Computational biology and bioinformatics, Genome informatics

## Abstract

*Kobresia* plants are important forage resources on the Qinghai-Tibet Plateau and are essential in maintaining the ecological balance of grasslands*.* Therefore, it is beneficial to obtain *Kobresia* genome resources and study the adaptive characteristics of *Kobresia* plants on the Qinghai-Tibetan Plateau. Previously, we have assembled the genome of *Carex littledalei* (*Kobresia littledalei*), which is a diploid with 29 chromosomes. In this study, we assembled genomes of *Carex parvula* (*Kobresia pygmaea*) and *Carex kokanica* (*Kobresia royleana*) via using Illumina and PacBio sequencing data, which were about 783.49 Mb and 673.40 Mb in size, respectively. And 45,002 or 36,709 protein-coding genes were further annotated in the genome of *C. parvula* or *C. kokanica.* Phylogenetic analysis indicated that *Kobresia* in Cyperaceae separated from Poaceae about 101.5 million years ago after separated from *Ananas comosus* in Bromeliaceae about 117.2 million years ago. *C. littledalei* and *C. parvula* separated about 5.0 million years ago, after separated from *C. kokanica* about 6.2 million years ago. In this study, transcriptome data of *C. parvula* at three different altitudes were also measured and analyzed. *Kobresia* plants genomes assembly and transcriptome analysis will assist research into mechanisms of plant adaptation to environments with high altitude and cold weather.

## Introduction

*Kobresia* plants (Cyperaceae) are the most important component of alpine grasslands on the Qinghai-Tibet Plateau^1^. *Kobresia* plants are important forage resources in alpine areas and are essential in maintaining the ecological balance of grasslands for their tolerance of cold, radiation, drought and strong wind^2^. The sedge *C. parvula* is the dominant species of high-altitude pastures in Tibet, and it is the most important source of forage in animal husbandry. It grows in alpine steppe, alpine meadow and swamp meadow on river beach, hillside, valley and terrace at an altitude of 3700–5300 m. *C. parvula* reaches only 2–3 cm in height, making it highly grazing tolerant. *C. kokanica* grows in moist grassland, swamp meadow and meadow grassland on hillsides, gullies, lakeside, alluvial fan and flood plain at an altitude of 3100–5200 m. *C. kokanica* reaches 10–60 cm in height and the morphological difference of this species is largely differed with altitudes.

Reference genomes of various individuals can provide new insights into genomic structure and evolution, genetic diversity, and phylogeny^3^. We have previously assembled the genome of *Carex littledalei*, which is a diploid with 29 chromosomes^4^. Basic chromosome numbers and ploidy are varied in different *Kobresia* species and difficult to verify, which take great challenge to the assembly of *Kobresia* genomes. Combined application of chromosome counts, new microsatellite markers and flow cytometry confirmed tetraploidy in *C. parvula* (2n = 4x = 64)^5^. Chromosome numbers of *Kobresia esenbeckii* (2n = 66), *Kobresia duthiei* (2n = 84), *Kobresia* curvata (2n = 50), *Kobresia schoenoides* (2n = 32) were reported and it is concluded that chromosomal evolution in the unispicate *Kobresia* species may have been caused by both polyploidy and aneuploidy^6^.

This study assembled and annotated genomes of *C. parvula* and *C. kokanica* based on long reads from the PacBio Sequel sequencing platform and short reads from the Illumina Hi-seq 2500 sequencing platform. The final genome assembly of *C. parvula* is approximately 783.49 Mb with a contig N50 of 468.079 kb. We predicted 45,002 protein-coding genes from the generated assembly of *C. parvula*, and 94.7% (42,630 genes) of all protein-coding genes were annotated. At the same time, genome assembly of *C. kokanica* is approximately 673.40 Mb with a contig N50 of 1.179 Mb. We predicted 36,709 protein-coding genes from the generated assembly of *C. kokanica*, and 96.6% (35,477 genes) of all protein-coding genes were annotated. The results of comparative genomic analysis have tentatively clarified the origin of the two species and revealed their genomic characteristics, which provide new insights for studies exploring genome evolution and reveal stress-specific genes of them.

## Data description

### Sample sequencing and genome size estimation

**Figure 1:**
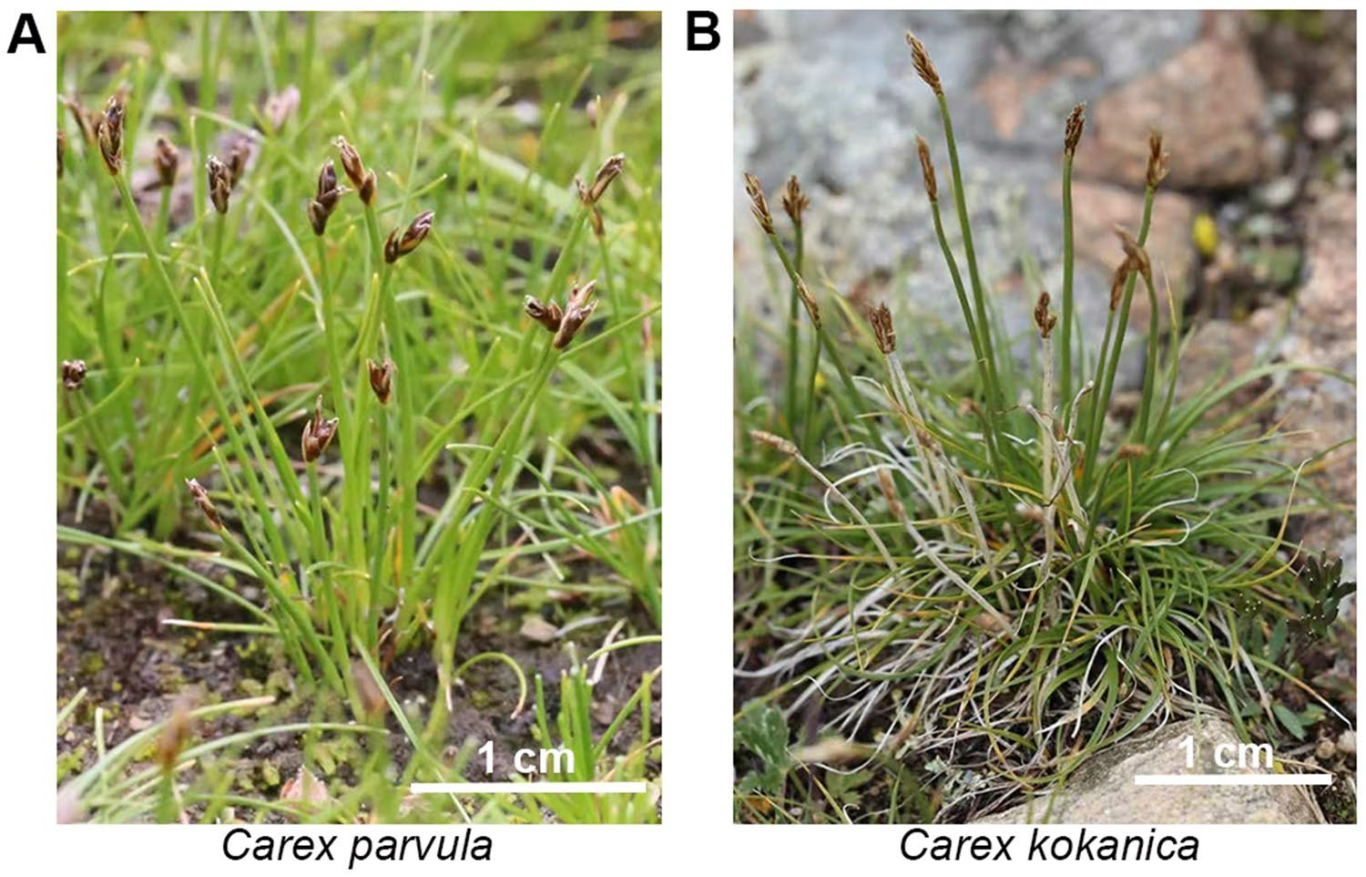
The *Carex parvula* and *Carex kokanica* used in this study. (**A**) The *C. parvula* individual. White bar = 1 cm. (**B**) The *C. kokanica* individual. White bar = 1 cm.

*C. parvula* (E: 91°59.7597′ N: 31°35.9755′) and *C. kokanica* (E: 91°10.5554′ N: 30°29.8873′) in anthesis stage were collected in July 2018 from Dangquka Village, Dangquka Town, Damxung County in the Tibet Autonomous Region of China. *C. parvula* and *C. kokanica* located at altitudes of up to 4600 m and 4275 m, respectively (Fig. 1). High-quality genomic DNA of *C. parvula* and *C. kokanica* was extracted from leaf tissue separately. Illumina’s Genomic DNA Sample Preparation kit was used for preparation of sequencing library. DNA library with insert sizes of 350 bp was sequenced on an Illumina HiSeq 2500 platform. 53.07 Gb and 55.31 Gb of short cleaned reads were generated for k-mer analysis and base correction, and their coverage were 137.59 × and 128.48 × of *C. parvula* and *C. kokanica* respectively (Supplemental Table S1, coverage were calculated based on genome size of haploid)^7^. The genome size was calculated based on k-mer (k = 17) statistics using the modified Lander–Waterman algorithm. As a result, the genome size of *C. parvula* was estimated to be 385.7 Mb (genome size of haploid) with 1.74% heterozygosity and a repeat sequence ratio of 47.97%, and *C. kokanica* was estimated to be 430.51 Mb (genome size of haploid) with 2.00% heterozygosity and a repeat sequence ratio of 47.84% (Supplemental Table S2, Supplemental Fig. S1). Meanwhile, we inferred that *C. parvula* is tetraploid and *C. kokanica* is triploid from k-mer analysis (Supplemental Fig. S1). Enriched large DNA fragments (> 10 kb) were sequenced on a PacBio Sequel system. 43.88 Gb and 44.25 Gb (113.77 × or 102.79 × coverage) of long sequencing reads were obtained after removing adaptors in polymerase reads of *C. parvula* and *C. kokanica,* with N50 length of 8219 bp and 15,090 bp respectively (Supplemental Table S1).

### Assembly of *C. parvula* and *C. kokanica* genome

First, pre-assembly reads were obtained by self-correcting of PacBio long reads and assembled into contigs by FALCON (falcon-kit = 0.7) through the “Overlap-Layout-Consensus” algorithm^8^. Contigs were corrected using PacBio long reads with quiver (smrtlink_6). Then, one round of polishing were applied to the assembled contigs using Pilon-1.18^9^ with the Illumina short reads. To improve the assembly of *C. parvula*, contigs were linked into scaffold using 93.27G 10 × data using FragScaff (PBSuite_15.8.24). The preliminary genome assembly of *C. parvula* includes 5261 contigs with N50 = 468,079 bp and longest scaffold = 7,682,949 bp. The genome is approximately 783.49 M in length and the GC content of the genome is 35.41% (Table 1). The preliminary genome assembly of *C. kokanica* includes 1504 contigs with N50 = 1,179,729 bp and longest scaffold = 4,432,967 bp. The genome is approximately 673.40 M in length and the GC content of the genome is 34.68% (Table 1). Compared with the estimated genome size, 1.56 (*C. kokanica*)—2.03 (*C. parvula*) times of genome sequences were assembled.

**Table 1:** Statistics of assembled *Carex parvula* and *Carex kokanica* assembly and annotation.

Genome assembly	*Carex parvula* (*Kobresia pygmaea*)	*Carex kokanica* (*Kobresia royleana)*
Number of scaffolds	5261	1504
Total length of scaffolds (bp)	783,486,840	673,403,748
N50 of scaffolds (bp)	468,079	1,179,729
Longest scaffold (bp)	7,682,949	4,432,967
GC content (%)	35.41	34.68
**Repeat annotation**		
Total (bp)	411,079,196(52.47%)	373,538,444(55.47%)
TRF (bp)	38,080,191(4.86%)	37,108,299(5.51%)
Transposable element (bp)	403,711,071(51.53%)	367,847,237(54.63%)
**Gene annotation**		
Number of genes	45,002	36,709
Total coding sequence length (bp)	154,945,486	124,438,004
Mean gene length (bp)	3443.08	3389.85
Mean number of exons per gene	5	5.73
Mean exon length (bp)	221.49	212.93
Average CDS length (bp)	1107.86	1220.16

### Repeat annotation of *C. parvula* and* C. kokanica* genome

We used LTR Finder^10^, RepeatScout (http://www.repeatmasker.org/) and RepeatModeler to identify ab initio repeat sequence library. Then RepeatMasker was used to predict repeat sequences of the genome through similarity searching of repetitive elements released by Repbase^11^ and our ab initio identified repeat sequence library. We also predicted repetitive elements by RepeatProteinMask and tandem repetitive sequences by TRF^12^. For *C. parvula*, a total of ~ 411.08 M repetitive elements including ~ 38.08 M tandem repetitive sequences and 403.71 M TE were identified, which was 52.46% of the genome (Supplemental Table S3). Among them, DNA transposons accounted for 18.60% of the genome. Retrotransposon including long terminal repeat (LTR), long interspersed nuclear elements (LINE) and short interspersed nuclear elements (SINE) accounted for 25.47%, 5.34% and 0.10% of the genome, respectively (Supplemental Table S4). For *C. kokanica*, we identified a total of ~ 373.54 M repetitive elements including ~ 37.11 M tandem repetitive sequences and 367.85 M TE, which was 55.47% of the genome (Supplemental Table S3). Among them, DNA transposons accounted for 26.06% of the genome. Retrotransposon including long terminal repeat (LTR), long interspersed nuclear elements (LINE) and short interspersed nuclear elements (SINE) accounted for 23.84%, 6.77% and 0.03% of the genome, respectively (Supplemental Table S4).

### Prediction and functional annotation of protein-coding genes

Ab initio, homology-based and RNA-seq-based pipelines were integrated for subsequent prediction of protein-coding genes of *C. parvula* genome and *C. kokanica* genome. SNAP^13^, Augustus^14^, Geneid^15^, GlimmerHMM^16^ and GENSCAN^17^ were used for ab initio gene prediction. For homology annotation, we used proteomes of *Zea mays*, *Setaria italica*, *Brachypodium distachyon*, *Ananas comosus, Oryza sativa* and *Arabidopsis thaliana* to predict protein-coding genes by GeneWise. Simultaneously, we used the RNA-seq data of 9 samples from different habitats and altitudes for *C. parvula* (HC26, HC27, HC28 (3381, alpine meadow), HC01, HC02, HC05 (4400, alpine meadow), HC04, HC59, HC60 (4250, Alpine swamp)) and 4 samples from different tissues (leaf, root, spike and stem) for *C. kokanica* to generate annotation results based on transcripts. After integrating results of the three sources of evidence by EVM, 58,702 (Supplemental Table S5) and 48,549 (Supplemental Table S6) primitive gene models were predicted for *C. parvula* and *C. kokanica* separately. We then filtered and polished these gene models through evidence number and expression level, and 45,002 genes in *C. parvula* and 36,709 genes in *C. kokanica* supported by at least two lines of evidence or with FPKM > 1 were retained. The average length of genes are 3443.08 bp and 3389.85 bp, while the average length of CDSs are 1107.86 bp and 1220.16 bp in *C. parvula* and *C. kokanica*, separately. There are 5 exons in each gene on average with length of 221.49 bp per exon in *C. parvula*, while there are 5.73 exons in each gene on average with length of 212.93 bp per exon in *C. kokanica* (Supplemental Table S7).

Functional annotation was obtained by mapping predicted protein sequences to KEGG^18^, SwissProt^19^ and Non-redundant protein NCBI databases^20^. Simultaneously, to inferring the functional annotation of protein coding genes by domain, the protein sequences were searched against member databases of Interpro using InterProScan^21–24^. Meanwhile, Gene Ontology (GO) terms were obtained by Blast2GO. Finally, a total of 44,796 (96.01%) out of 45,002 genes have integrated functional annotation in *C. parvula*, and a total of 36,579 (97.40%) out of 36,709 genes have integrated functional annotation in *C. parvula* (Supplemental Table S8). In *C. parvula*, 30,282 (67.3%) genes have blast hit in all four databases, and 26,267 (71.5%) genes of C. kokanica have blast hit in all four databases (Supplemental Fig. S2). Gene sets were assessed with BUSCO Version 3.0.2^25^, in which 85.9% and 88.2% complete gene models of 1440 embryophyta core genes (odb9) were identified in *C. parvula* and *C. kokanica*, respectively (Supplemental Table S9). We also compared the colinear gene pairs between *C. parvula/C. kokanica* and *C. littledalei.* There are 8926 and 4010 genes have 1:1 ortholog to *C. littledalei in C. parvula* and *C. kokanica* respectively. And There are 4437 and 10,061 genes have 1:2 ortholog to *C. littledalei in C. parvula* and *C. kokanica* respectively (Supplemental Table S10). We also illustrated the synteny between contigs of *C. parvula*, *C. kokanica* and *C. littledalei* Chromosome 2 (Supplemental Fig. S3). The comparison between *C. kokanica* and *C. littledalei* clearly revealed the 2:1 collinear relationship. However, the comparison between *C. parvula* and *C. littledalei* didn’t show remarkable 2:1 collinearity, which is likely due to the comparatively shorter contigs.

### Comparative genome analysis and divergence time estimation

**Figure 2:**
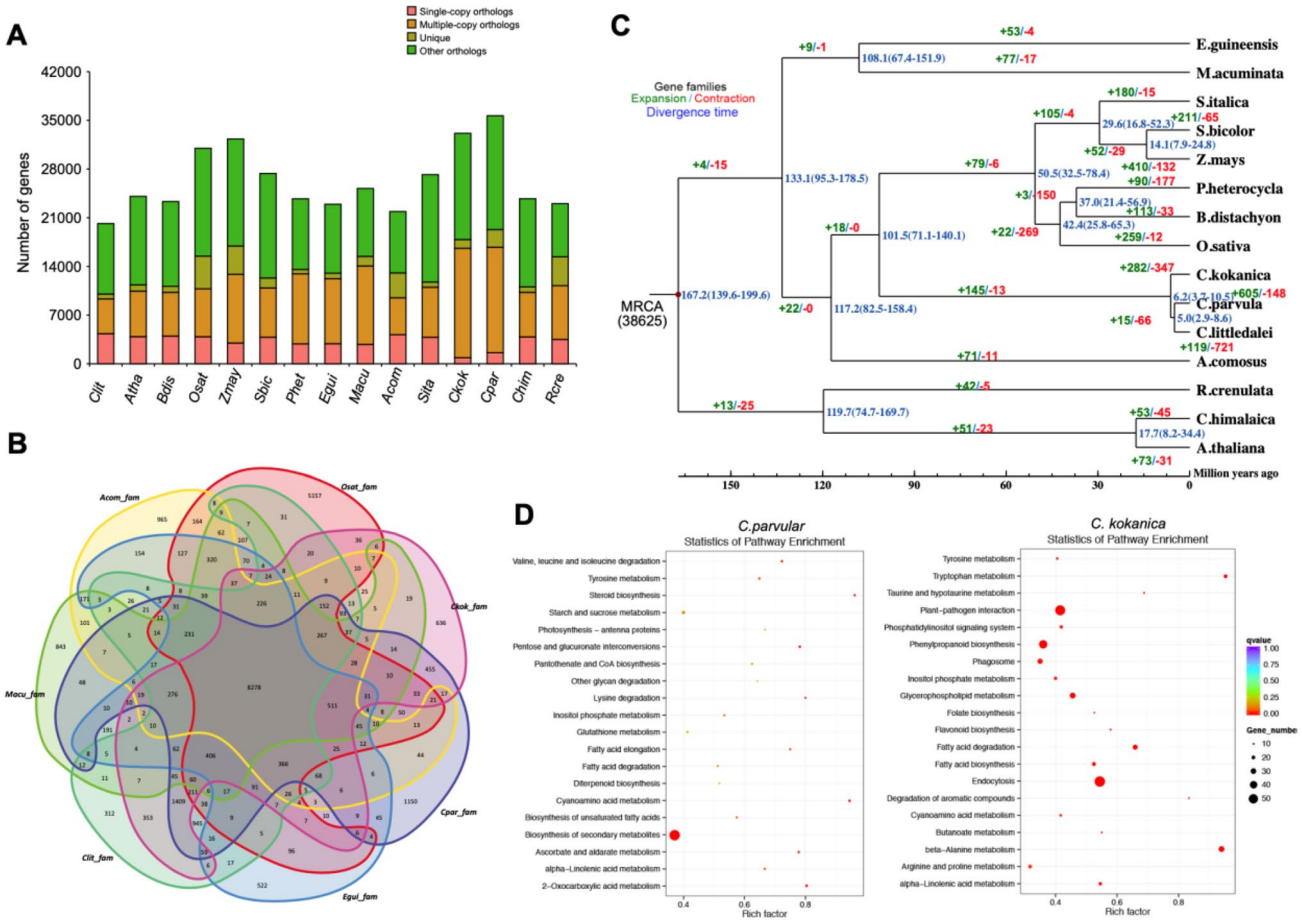
Comparative analyses of *Carex parvula* and *Carex kokanica* with other plants. (**A**) The distribution of genes (single-copy genes, multiple-copy genes, unique genes, other and uncluster genes number) in 15 different species. *Carex littledalei* (Clit), *Arabidopsis thaliana* (Atha), *Brachypodium. Distachyon* (Bdis), *Oryza sativa* (Osat), *Zea mays* (Zmay), Sorghum. Bicolor (Sbic), *Phyllostachys heterocycle* (Phet), *Elaeis guineensis* (Egui), *Musa acuminate* (Macu), *Ananas comosus* (Acom), *Setaria italica* (Sita), *Carex kokanica* (Ckok), *Carex parvula* (Cpar), *Crucihimalaya himalaica* (Chim), *Rhodiola crenulate* (Rcre). (**B**) Common and unique gene families between Acom, Osat, Macu, Clit, Egui, Cpar and Ckok*.* (**C**) Gene family expansions and contractions and estimation of divergence time in *Carex parvula*, *Carex kokanica* and 13 other species. (**D**) Enriched KEGG terms of the expanded genes of *C. parvula* and *C. kokanica* (*K. royleana*).

The assembly of *C. kokanica, C. parvula* and *C. littledalei* genomes provides an opportunity to resolve the relationships among *Kobresia* plants*.* Using 3 eudicots (*Arabidopsis thaliana, Crucihimalaya himalaica *and* Rhodiola crenulate*) and 12 monocots (*Brachypodium Distachyon*, *Oryza sativa*, *Zea mays*, *Sorghum bicolor*, *Phyllostachys heterocycle*, *Elaeis guineensis*, *Musa acuminate*, *Ananas comosus*, *Setaria italica*, *C. kokanica, C. parvula and C. littledalei)*, we identified 38,632 gene families including 50 single-copy orthologues using OrthoMCL^26^. A total of 33,136 or 35,668 of *C. kokanica* or *C. parvula* genes were clustered into four groups and included 1255 or 2556 unique genes, 875 or 1584 single-copy orthologs, respectively (Fig. 2A). And compared to *A. comosus*, *O. sativa*, *M. acuminate*, *E. guineensis* and *C. littledalei,* 636 gene families were specific in the *C. kokanica* genome and 1150 gene families were specific in the *C. parvula* genome (Fig. 2B). A Maximum likelihood phylogeny was inferred by RAxML^27^ with concatenated alignments of 50 single-copy orthologues. Further divergence time of each species was estimated by MCMCtree in Phylogenetic Analysis by Maximum Likelihood (PAML), *Kobresia* in *Cyperaceae* separated from *Poaceae* about 101.5 million years ago after separating from *Ananas comosus* in Bromeliaceae about 117.2 million years ago, *C. littledalei* and *C. parvula* separated about 5.0 million years ago (MYA), after separated from *C. kokanica* about 6.2 MYA (Fig. 2C), and the time correction points were taken from the TimeTree website.

CAFE (https://sourceforge.net/projects/cafehahnlab) were used to conduct the gene family expansion/contraction analysis^28^. 605 expanded and 148 contracted gene families were discovered in the genome of *C. parvula*, and the expanded gene families were enriched in transport, localization and oxidation–reduction biological processes with the molecular function of catalytic activity et al. But in *C. kokanica* genome, 282 expanded and 347 contracted gene families were discovered. The expanded gene families were significantly enriched in metabolic and oxidation–reduction biological processes with the molecular function of catalytic activity, ion binding et al. (Supplemental Fig. S3). Oxidation–reduction biological processes plays an important role in the response of plants to stress, and it is very important for these two *Kobresia* plants to adapt to the plateau environment. However, there was no obvious enrichment result in the constricted genome families of these two genomes. But it is worth noticing that although the results of GO enrichment analysis were relatively similar, the results of KEGG pathway enrichment of these two genomic expansion genes were significantly different (Fig. 2D). Expanded gene families of *C. parvula* were enriched in the cyanoamino acid metabolism (*p*-value 9.94E−07), ascorbate and aldarate metabolism (*p*-value 9.62E−05) et al. while that of *C. kokanica* were significantly enriched in the metabolism of beta-Alanine (*p*-value 6.08E−12), tryptophan (*p*-value 3.72E−08) and glycerophospholipid (*p*-value 1.73E−06) etc. The results preliminarily revealed that the two *Kobresia* plants had different preferences in the process of evolution and environmental adaptation.

The branch-site model in PAML only detected 5 gene families were positively selected in *C. kokanica*^29^, namely, GTP-binding protein OBGC1 (required for chloroplast development)^30^, metallo-beta-lactamase superfamily, ABC transporter (regulation of chlorophyll biosynthetic process)^31^, one cold and water deprivation response ATPase (AAA family)^32^ and Histone-lysine N-methyltransferase ASHH1 (paly roles in DNA repair)^33^. Positive selection for these genes coincided with conditions such as extreme weather at high altitudes and intense UV exposure.

### Global transcriptome analysis of *C. parvula* at different altitudes

**Figure 3:**
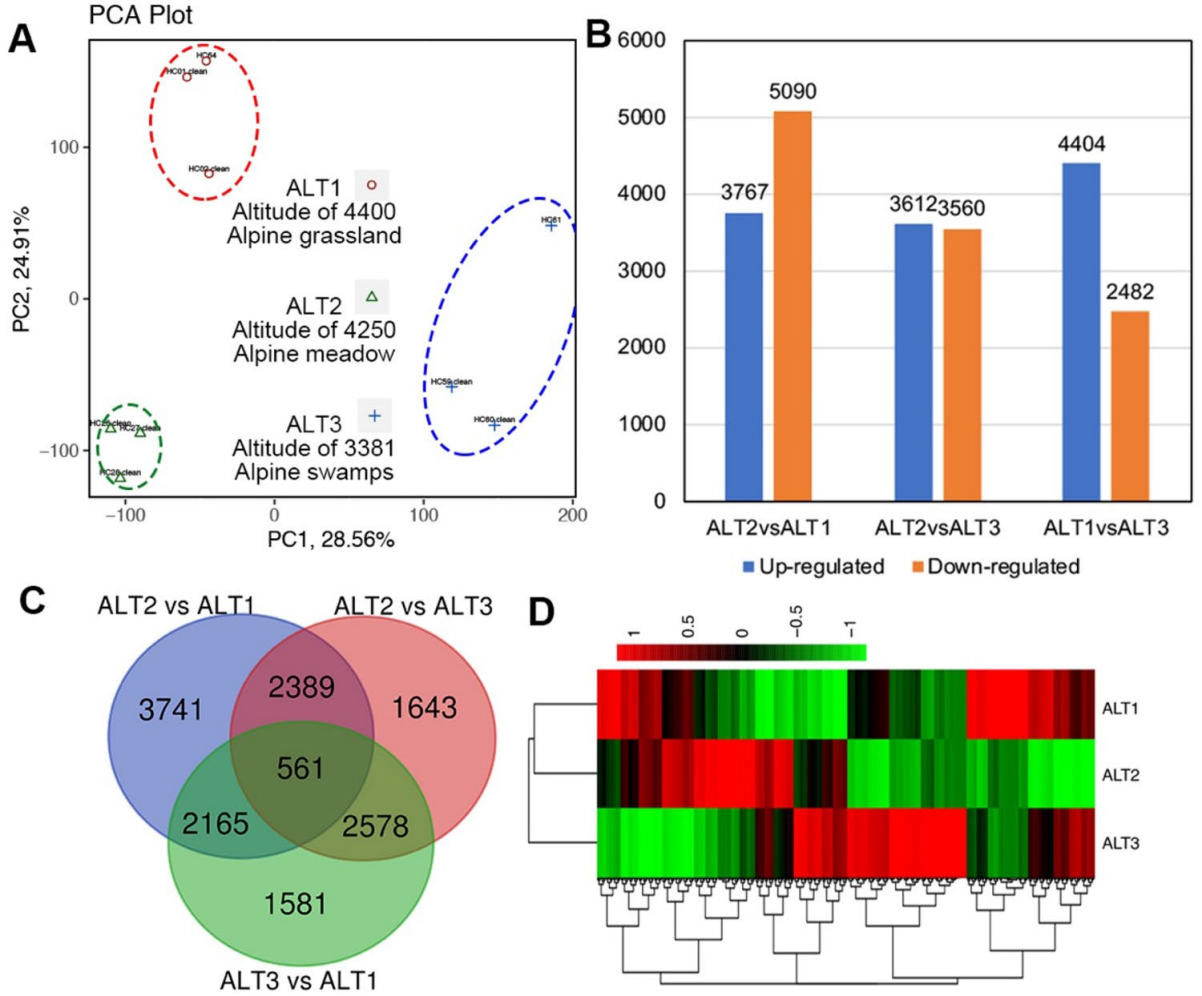
Overall differentially expressed genes (DEGs) of *Carex parvula* from different altitudes and environments. (**A**) Principal component analysis (PCA) of differentially expressed genes (log2 (relative expression level) ≥ 1 and ≤  − 1, DEGs) in 3 groups. (**B**) The histogram of the statistical results for the DEGs in 3 groups. The orange for down-expressed genes and the blue for up-expressed genes. (**C**) Venn diagram of the overlap between the DEGs between ALT2 vs ALT1, ALT2 vs ALT3 and ALT3 vs ALT1. D, The clustering analysis results of the relative expression levels of the different groups.

Transcriptome analysis of *Kobresia* plants at different altitudes can provide a crucial systems-level insight into the molecular mechanisms underlying the mechanisms of plant adaptation to environments with high altitude and cold weather. Three representing materials, at altitudes of 4400 m (Alpine grassland), 4250 m (Alpine meadow) and 3381 m (Alpine swamp), were chosen and respectively named ALT1, ALT2 and ALT3 with three biological duplicates. Differentially expressed genes (log2 (relative expression level) ≥ 1 and ≤ − 1, padj ≤ 0.05, DEGs) between ALT2 vs ALT1, ALT2 vs ALT3 and ATL3 vs ALT1 were identified by RNA-Seq (Supplemental Tables S11/S12/S13). Principal component analysis (PCA) of the DEGs revealed that the separation of gene expression profiles among these three groups were quite different from each other, which proved the representativeness of these material (Fig. 3A). 3767 upregulated and 5090 downregulated genes were observed in ALT2 compared with ALT1, 3612 upregulated and 3560 downregulated genes were observed in ALT2 against ALT3, and 4404 upregulated and 2482 downregulated genes were observed in ALT1 compared with ALT3 (Fig. 3B). Venn diagram showed that only 561 genes were shared, but a large number of specific differentially expressed genes were identified among these three comparable groups (Fig. 3C). The clustering analysis results of the relative expression levels of the overlapped genes in the three groups are also shown in Fig. 3D, which also proved the representative of data.

### Transcriptome differences in photosynthesis and stress response

**Figure 4:**
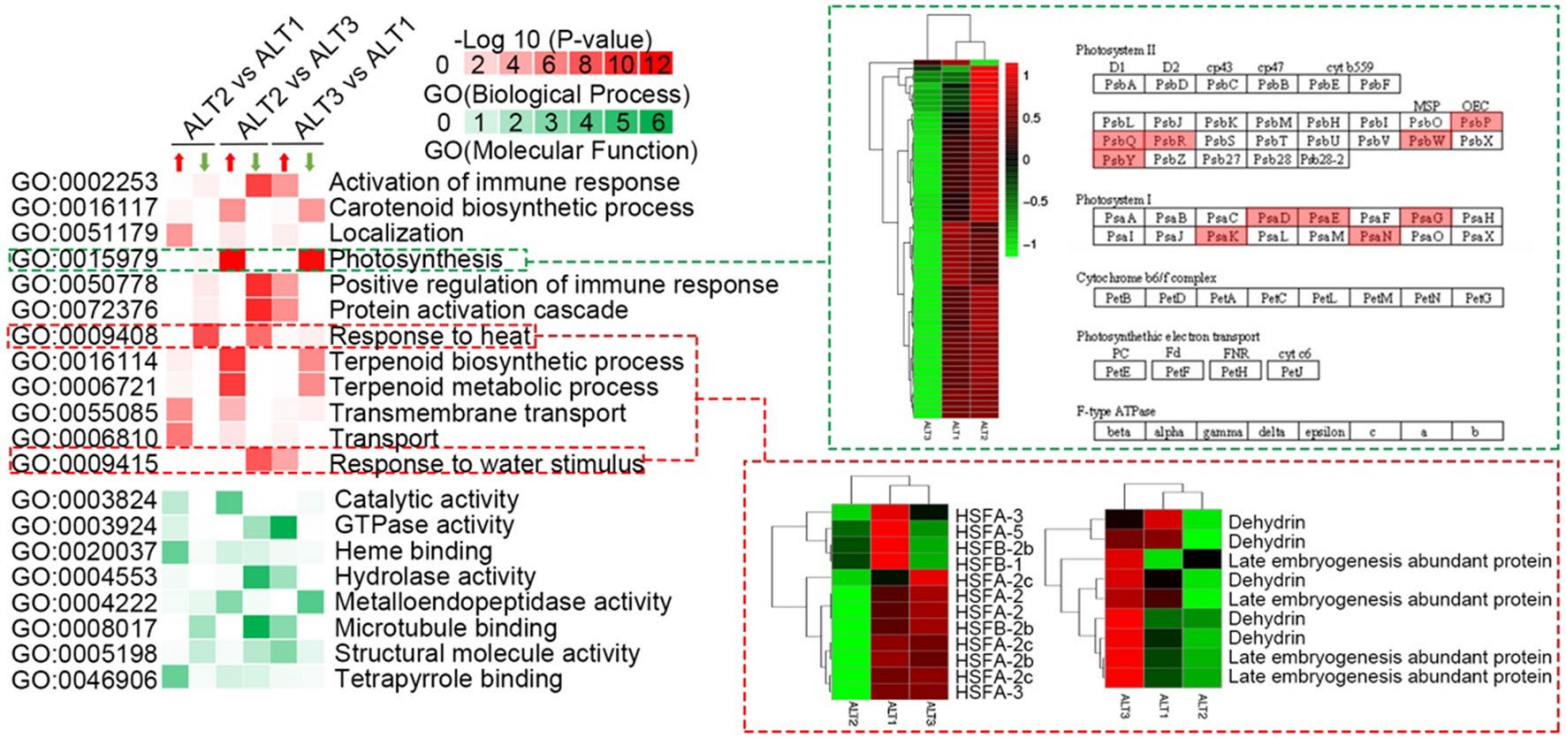
DEGs in photosynthesis and stress response. GO enrichment analysis clustering results (red for the biological process; green for the molecular function) of the up-expressed and down-expressed genes. The clustering analysis results of genes, which belonged to the photosynthesis, response to heat and water stimulus, relative expression levels of these three different groups based on the results of RNA-seq analysis.

Gene Ontology (GO) enrichment analyses were performed with the DEGs between ALT1, ALT2 and ALT3 (Fig. 4). By comparison, genes in photosynthesis (GO:0015979), response to heat (GO:0009408) and response to water stimulus (GO:0009415) were significantly enriched. 9 of the 12 heat stress transcription factors (HSF) had lower expression level in ALT2 when compared with ALT1 and ALT3. The expression of five dehydrins (cold-responsive genes)^34^ was also the lowest in the ALT2 and showed the similar expression trend with HSF (Fig. 4, red dotted line). All of these data were just consistent with the location of ALT2 in the alpine meadow with relatively higherwater content than ALT1 and higher altitude than ALT3. The clustering analysis results of the expression levels of all the DEGs involved in photosynthesis were shown in Fig. 4, and the expression patterns were significantly lower in ALT3. These differences involved PsbP/Q/R (23/16/10 kDa subunit of oxygen evolving system)^35–37^, PsbW (stabilizes dimeric photosystem II)^38^, PsbY (core for cell redox homeostasis)^39^ of the photosystem II reaction center, and PsaD/E (reaction center subunit II to effect the stability of PS I)^40^, PsaG (subunit V, plays an important role in electron transport and the PS stabilization)^41,42^, PsaK (subunit X, effects on photosynthetic electron flow)^43^ and PsaN (involves in the interaction between plastocyanin and PS I)^44^ of photosystem I reaction center. In general, by comparing leaf transcriptome data in three habitats with different altitudes and different water contents, it can be found that *C. parvula* can respond to different temperature and water conditions by regulating HSF, dehydrating proteins, etc., and respond to different light conditions by affecting the stability of PS II and PS I, etc.

## Conclusion

In this study, we presented the scaffold-level genomes of *Carex parvula* and *Carex kokanica* and described their genetic attributes. Comparative genomic analysis revealed their evolutionary status and the preference of genes positive selection in the genus *Kobresia* under the plateau environment stress. The difference analysis of transcriptome data of *C. parvula* at different altitudes and habitats further revealed mechanisms of plant adaptation to environments with high altitude and cold weather. This study provides a valuable reference for further study on genomic evolution, preservation and utilization of excellent traits of *Kobresia* plants.

## Methods

### Sample collection, DNA extraction and sequencing

*C. parvula* (E: 91°59.7597′ N:31°35.9755′) and *C. kokanica* (E: 91°10.5554′ N:30°29.8873′) were collected in July 2018 from public land of Dangquka Village, Dangquka Town, Damxung County in the Tibet Autonomous Region of China. Guangpeng Qu undertook the formal identification of the plant material. All collected specimens were submitted to the herbaria of Tibet Academy of Agriculture and Animal Husbandry Science, Lhasa, Tibet, China. The deposition number of *C. parvula* is 1807002, and the deposition number of *C. kokanica* is 1807003.

For Illumina sequencing, Genomic DNA was extracted from the leaf tissues of all samples using the CTAB method. DNA from each sample was randomly fragmented by nebulization to an average size of 350 bp and processed by the Illumina DNA sample preparation protocol. The DNA library was prepared according to the instructions of the manufacturer and sequencing was performed on the Illumina HiSeq 2500 platform (paired-end 2 × 150 bp).

For Pacbio sequencing, a total of 10 μg of sheared DNA was used for a 20-kb insert size library construct and the DNA library were sequenced on the PacBio RS II platform.

For 10× sequencing, DNA sample preparation, indexing, and barcoding were done using the GemCode Instrument from 10× Genomics. About 0.7 ng input DNA with 50 kb length was used for GEM reaction procedure during PCR, and 16-bp barcodes were introduced into droplets. Then, the droplets were fractured following the purifying of the intermediate DNA library. Next, we sheared DNA into 500 bp for constructing libraries, which were finally sequenced on the Illumina HiseqXTen.

### Genome assembly

To estimate the genome size of *C. parvula* and *C. kokanica*, we used reads from paired-end libraries to determine the distribution of K-mer values. According to the Lander–Waterman theory, genome size can be determined by the total number of K-mers divided by the peak value of the K-mer distribution. Given the number of peaks in the K-mer distribution of *C. parvula* and *C. kokanica*, it was indicated that *C. parvula* is tetraploid and *C. kokanica* is triploid. The estimated ploidy of *C. parvula* is consistent with the report of Seeber et al.^5^. With the major peak at the expected K-mer depth and the formula genome size = total K-mer/expected K-mer depth, the size of the haploid genome of *C. parvula* and *C. kokanica* was estimated to be 396.40 M and 447.68 M.

For genome assembly, pre-assembly reads were obtained by self-correcting of PacBio long reads and assembled into contigs by FALCON (falcon-kit = 0.7) through the “Overlap-Layout-Consensus” algorithm^8^. Conitgs were corrected using PacBio long reads with quiver (smrtlink_6). Then, using Illumina short reads to improve the precision in Pilon-1.18^9^. To improve the assembly of C. parvula, contigs were linked into scaffold using 10× data using FragScaff (PBSuite_15.8.24).

### Genome annotation

Transposable elements in the assembly were identified both at DNA and protein levels. We used RepeatModeler, RepeatScout and LTRFinder^10^ to de novo identify and classify repeated sequences in each genome. RepeatMasker was applied for DNA-level identification using Repbase^11^ and the de novo transposable element library. At the protein level, RepeatProteinMask was used to searches against the transposable element protein database. Overlapping transposable elements belonging to the same type of repeats were merged.

Ab initio, homology-based and RNA-seq-based pipelines were integrated for subsequent prediction of protein-coding genes of *C. parvula* genome and *C. kokanica* genome. SNAP^13^, Augustus^14^, Geneid^15^, GlimmerHMM^16^ and GENSCAN^17^ were used for ab initio gene prediction. For homology annotation, we used proteomes of *Zea mays*, *Setaria italica*, *Brachypodium distachyon*, *Ananas comosus*, *Oryza sativa* and *Arabidopsis thaliana* to predict protein-coding genes by GeneWise. Simultaneously, we used the RNA-seq data from 10 samples from different habitats and altitudes for *C. parvula* (HC26, HC27, HC28 (3381, alpine meadow), HC01, HC02, HC05 (4400, alpine meadow), HC04, HC59, HC60 (4250, Alpine swamp) and 4 sample from different tissues (leaf, root, spike and stem) for *C. kokanica* to generate annotation results based on transcripts.

Functional annotation was obtained by mapping predicted protein sequences to KEGG^18^, SwissProt and Non-redundant protein NCBI databases for plants. Simultaneously, to inferring the functional annotation of protein coding genes by domain, the protein sequences were searched against member databases of InterPro^23^ using InterProScan^24^. Meanwhile, Gene Ontology (GO) terms were obtained by Blast2GO.

### Comparative genomics and divergence time estimation

Orthologous groups of selected plant species were constructed using OrthoMCL^26^. Protein sequences of each single-copy orthologous group were aligned with MAFFT (v.7.313)^45^ with parameters ‘--maxiterate 1000 --localpair’. Alignments of resulting orthologous groups were concatenated to build a maximum likelihood phylogeny with RAxML. The divergence time was estimated using MCMCTree with branch lengths estimated by BASEML in the PAML package^46^ with calibration point of Angiospermae (168–194 Mya).

For gene family evolution analysis, modeling of gene family size was performed by CAFE (v4.2)^28^. Orthologous groups with family-wide *p* < 0.05 were defined as rapidly evolving gene families whereas, the Viterbi *p* (< 0.05) was used to identify branches with gene families significantly expanded or contracted compared with their last common ancestor.

### Sample collection, RNA extraction and sequencing

Three representing materials, altitude of 4400 m (ALT1, Alpine grassland, Dangxiong, Tibet), 4250 m (ALT2, Alpine meadow, Dangxiong, Tibet) and 3381 m (ALT3, Alpine swamp, Qinghai) were collected from public land. We collected three samples in each site. Fresh leaves were stored in liquid nitrogen and transported to Novogene (Tianjin, China) for subsequent procedures. Total RNA of leaves of each sample was extracted with NEB Next^®^ Ultra™ RNA Library Prep Kit, sequenced by Illumina HiSeq2500.

### Differentially expressed gene analysis

RNA-seq reads were trimmed by Trimmomatic and then mapped to the *C. parvula* genome by HiSAT2^47^. FPKM was calculated on the basis of unique mapping reads using the HTseq package. Different expressed genes (Foldchange > 2 and *p* value < 0.05) were identified using cuffdiff in cufflinks-1.3.0^48^.

We confirm that all methods were performed in accordance with the relevant guidelines and regulations.

## Supplementary Information

Supplementary Information 1.

Supplementary Information 2.

Supplementary Information 3.

Supplementary Information 4.

## Data Availability

We have uploaded our raw data and assemblies into NCBI with project accession ID: PRJNA734565 (*C. kokanica*) and PRJNA731336 (*C. parvula*).
