# Supplementary material for: Draft genomes assembly and annotation of Carex parvula and Carex kokanica reveals stress-specific genes
Source: Sci Rep. 2022 Mar 23;12:4970. doi: 10.1038/s41598-022-08783-z (PMC8943043; doi:10.1038/s41598-022-08783-z)
Supplement: Supplementary file 1 — Supplementary Information 1. [file 41598_2022_8783_MOESM1_ESM.docx]

**Supplemental Table S1. Sequencing data used for *Carex parvula* and *Carex kokanica* genome assembly.**

The coverage was calculated using the total length of scaffolds.

| **Library types** | **Insert size (bp)** | **Total data (Gb)** | **Read length (bp)** | **Sequence coverage (X)** |
| --- | --- | --- | --- | --- |
|  |  | ***Cpar/ Ckok*** |  | ***Cpar/ Ckok*** |
| Illumina reads | 350 | 53.07/ 55.31 | 150 | 137.59/128.48 |
| Pacbio reads | 20,000 | 43.88/44.25 | 8,219*/15,090* | 113.77/102.79 |
| 10X |  | 93.27 /- |  | 235.29/- |
| Total | — | 190.22/ 99.55 | - | 486.65/231.27 |

* Read length(N50) of Pacbio subreads.

**Supplemental Table S2. Statistics of Survey.**

| **species** | **Kmer** | **Depth** | **n_kmer** | **Genome size(M)** | **Revised Genome size(M)** | **Heterozygous rate(%)** | **Repeat rate(%)** |
| --- | --- | --- | --- | --- | --- | --- | --- |
| *Carex parvula* | 17 | 107 | 42,414,966,545 | 396.40 | 385.70 | 1.74 | 47.97 |
| *Carex kokanica* | 17 | 106 | 47,454,218,802 | 447.68 | 430.51 | 2.00 | 47.84 |

**Supplemental Table S3. Summary of Repeat contents in the *Carex parvula* and *Carex kokanica* genome**

| **Species** | ***C. parvula*** | ***C. kokanica*** |
| --- | --- | --- |
| Type | Repeat Size(bp) | % of genome |
| TRF | 38,080,191 | 4.860349 |
| RepeatMasker | 396,472,914 | 50.603647 |
| RepeatProteinMask | 71,206,236 | 9.088377 |
| Total | 411,079,196 | 52.467913 |

**Supplemental Table S4. Summary of TE contents in the *Carex parvula* and *Carex kokanica* genome**

The statistical results do not include TRF identification results. Denovo+Repbase is predicted using RepeatModeler, RepeatScout, and LTR_FINDER software; TE Proteins are transposons obtained by RepeatProteinMask against RepBase protein library; Combined TEs is the result obtained by the above categories, and the non-redundant result after removing the overlapped parts between them.

| Type | Species | Denovo+Repbase | | TE Proteins | | Combined TEs | |
| --- | --- | --- | --- | --- | --- | --- | --- |
|  |  | Length  (bp) | %in Genome | Length  (bp) | % in Genome | Length  (bp) | % in Genome |
| DNA | *C. parvula* | 139,116,831 | 17.756116 | 19,938,107 | 2.544792 | 145,712,936 | 18.598007 |
|  | *C. kokanica* | 172,696,971 | 25.645383 | 14,998,219 | 2.227225 | 175,507,457 | 26.062738 |
| LINE | *C. parvula* | 34,266,596 | 4.373602 | 14,033,789 | 1.791197 | 41,823,460 | 5.338119 |
|  | *C. kokanica* | 39,450,787 | 5.858415 | 11,192,367 | 1.662059 | 45,585,422 | 6.769404 |
| SINE | *C. parvula* | 818,190 | 0.104429 | 0 | 0 | 818,190 | 0.104429 |
|  | *C. kokanica* | 191,394 | 0.028422 | 0 | 0 | 191,394 | 0.028422 |
| LTR | *C. parvula* | 198,602,341 | 25.348523 | 37,292,650 | 4.759831 | 199,529,228 | 25.466826 |
|  | *C. kokanica* | 159,673,094 | 23.711346 | 26,642,155 | 3.956342 | 160,511,878 | 23.835905 |
| Unknown | *C. parvula* | 38,515,866 | 4.915956 | 49,806 | 0.006357 | 38,564,352 | 4.922144 |
|  | *C. kokanica* | 18,569,525 | 2.757562 | 38,295 | 0.005687 | 18,607,820 | 2.763249 |
| Total | *C. parvula* | 396,472,914 | 50.603647 | 71,206,236 | 9.088377 | 403,711,071 | 51.527486 |
|  | *C. kokanica* | 362,422,644 | 53.819517 | 52,824,710 | 7.844434 | 367,847,237 | 54.625065 |

**Supplemental Table S5. Basic statistical results of gene structure prediction of *Carex parvula* genome**

* Contains UTR areas, the rest are not

| **Gene set** | | **Number** | **Average gene length (bp)** | **Average CDS length (bp)** | **Average exons per gene** | **Average exon length (bp)** | | **Average intron length (bp)** |
| --- | --- | --- | --- | --- | --- | --- | --- | --- |
| ***De novo*** | Augustus | 54,637 | 2,654.97 | 1,046.23 | 4.23 | | 247.29 | 497.96 |
|  | GlimmerHMM | 98,601 | 6,355.58 | 651.15 | 3.08 | | 211.12 | 2,736.91 |
|  | SNAP | 101,117 | 4,743.75 | 724.97 | 4.89 | | 148.2 | 1,032.58 |
|  | Geneid | 44,814 | 7,473.26 | 848.14 | 5.11 | | 166.03 | 1,612.6 |
|  | Genscan | 51,231 | 8,935.42 | 1,169.6 | 5.6 | | 208.77 | 1,687.36 |
| **Homolog** | *Oryza_sativa* | 53,977 | 2,127.26 | 1,013.64 | 3.48 | | 291.66 | 449.87 |
|  | *Setaria_italica* | 47,089 | 2,429.15 | 1,002.08 | 3.99 | | 251.14 | 477.26 |
|  | *Arabidopsis_thaliana* | 47,818 | 2,312.72 | 987.41 | 3.74 | | 263.88 | 483.35 |
|  | *Zea_mays* | 45,200 | 2,633.51 | 1,113.71 | 3.97 | | 280.25 | 511.03 |
|  | *Brachypodium_distachyon* | 49,671 | 2,451.07 | 1,125.78 | 3.86 | | 291.76 | 463.61 |
|  | *Ananas_comosus* | 44,803 | 3,216.07 | 1,021.37 | 4.12 | | 247.84 | 703.19 |
| **RNA-seq** | Cufflinks | 107,036 | 8,442.86 | 1,716.05 | 5.12 | | 335.36 | 1,633.91 |
|  | PASA | 88,698 | 3,549.26 | 928.7 | 4.67 | | 198.73 | 713.42 |
| **EVM** | | 58,702 | 3,041.62 | 1,007.82 | 4.34 | | 231.95 | 608.02 |
| **PASA-update** | | 58,456 | 2,992.2 | 1,015.58 | 4.33 | | 234.32 | 592.85 |
| **Final set** | | 45,002 | 3,443.08 | 1,107.86 | 5 | | 221.49 | 583.53 |

**Supplemental Table S6. Basic statistical results of gene structure prediction of *Carex kokanica* genome**

* Contains UTR areas, the rest are not

| **Gene set** | | **Number** | **Average gene length (bp)** | **Average CDS length (bp)** | **Average exons per gene** | **Average exon length (bp)** | | **Average intron length (bp)** |
| --- | --- | --- | --- | --- | --- | --- | --- | --- |
| ***De novo*** | Augustus | 46,272 | 2,696.71 | 1,085.26 | 4.85 | | 223.66 | 418.31 |
|  | GlimmerHMM | 73,051 | 7,137.55 | 726.21 | 3.48 | | 208.79 | 2,587.08 |
|  | SNAP | 75,097 | 3,832.69 | 790.51 | 5.12 | | 154.53 | 739.16 |
|  | Geneid | 35,257 | 8,331.02 | 882.91 | 5.71 | | 154.71 | 1,582.44 |
|  | Genscan | 40,050 | 9,996.72 | 1,238.03 | 6.19 | | 199.85 | 1,686.06 |
| **Homolog** | *Oryza_sativa* | 43,704 | 2,587.9 | 1,204.61 | 4.06 | | 296.46 | 451.56 |
|  | *Setaria_italica* | 40,470 | 3,189.57 | 1,062.72 | 4.34 | | 245.01 | 637.28 |
|  | *Arabidopsis_thaliana* | 43,224 | 2,415.23 | 1,020.61 | 3.89 | | 262.38 | 482.6 |
|  | *Zea_mays* | 41,368 | 2,662.21 | 1,163.03 | 4.1 | | 283.83 | 483.98 |
|  | *Brachypodium_distachyon* | 48,348 | 2,221.15 | 1,080.92 | 3.61 | | 299.73 | 437.49 |
|  | *Ananas_comosus* | 41,154 | 2,570.78 | 1,051.19 | 4.26 | | 246.55 | 465.63 |
| **RNA-seq** | Cufflinks | 53,602 | 4,587 | 1,693.15 | 5.57 | | 303.81 | 632.8 |
|  | PASA | 62,172 | 2,806.05 | 1,002.01 | 5.33 | | 188.02 | 416.71 |
| **EVM** | | 48,549 | 2,965.18 | 1,079.05 | 4.96 | | 217.54 | 476.27 |
| **PASA-update** | | 48,296 | 2,937.61 | 1,089.39 | 4.98 | | 218.96 | 464.93 |
| **Final set** | | 36,709 | 3,389.85 | 1,220.16 | 5.73 | | 212.93 | 458.68 |

**Supplemental Table S7. The statistical results of gene structure**

| **Species** | **Number** | **Average gene length (bp)** | **Average CDS length (bp)** | **Average exons per gene** | **Average exon length (bp)** | **Average intron length (bp)** |
| --- | --- | --- | --- | --- | --- | --- |
| *Carx parvula* | 45,002 | 3,443.08 | 1,107.86 | 5 | 221.49 | 583.53 |
| *Carex kokanica* | 36,709 | 3,389.85 | 1,220.16 | 5.73 | 212.93 | 458.68 |
| *Oryza_sativa* | 34,227 | 2,205.44 | 1,003.17 | 3.83 | 262.06 | 425.13 |
| *Ananas_comosus* | 27,024 | 4,341.8 | 1,171.28 | 5.53 | 211.68 | 699.41 |
| *Setaria_italica* | 35,471 | 2,216.59 | 1,117.87 | 4.39 | 254.45 | 323.8 |
| *Brachypodium_distachyon* | 26,552 | 2,852.42 | 1,284.24 | 5.02 | 255.71 | 389.87 |
| *Zea_mays* | 38,985 | 3,488.05 | 1,185.11 | 4.95 | 239.66 | 583.76 |
| *Arabidopsis_thaliana* | 35,386 | 1,947.9 | 1,230.62 | 5.57 | 220.87 | 156.9 |

**Supplemental Table S8. The statistical results of gene function annotation of *Carex parvula* and *Carex kokanica* genome**

|  | | ***C. parvula*** | | ***C. kokanica*** | |
| --- | --- | --- | --- | --- | --- |
| **Database** | | **Annotated Num** | **Annotated Percent(%)** | **Annotated Num** | **Annotated Percent(%)** |
| **NR** | | 42,630 | 94.7 | 35,477 | 96.6 |
| **Swiss-Prot** | | 35,310 | 78.5 | 29,847 | 81.3 |
| **KEGG** | | 32,346 | 71.9 | 27,858 | 75.9 |
| **InterPro** | All | 38,708 | 86.01 | 32,569 | 88.72 |
|  | Pfam | 34,915 | 77.59 | 29,951 | 81.6 |
|  | Blast2GO | 31,844 | 70.76 | 26,890 | 73.25 |
| **Annotated** | | 44,796 | 96.01 | 36,579 | 97.40 |
| **Total** | | 45,002 | - | 36,709 | - |

**Supplemental Table S9. The estimation of the completeness for *C. parvula* and *C. kokanica* genome assembly based on BUSCO**

| Genome | BUSCO notation assessment results |
| --- | --- |
| *C. parvula* | C*:85.9% [S*:35.2%, D*:50.7%], F*:3.9%, M*:10.2%, n*:1440 |
| *C. kokanica* | C*:88.2% [S*:20.6%, D*:67.6%], F*:1.9%, M*:9.9%, n*:1440 |

*C：Complete BUSCOs, S：Complete and single-copy BUSCOs, D：Complete and duplicated BUSCOs, F：Fragmented BUSCOs, M：Missing BUSCOs, n：Total BUSCO groups searched

**Supplemental Table S10. Gene pairs compared *C. parvula and C. kokanica* with *C. littledalei***

|  | *C. parvula* | *C. kokanica* |
| --- | --- | --- |
| 1:1* | 8926 | 4010 |
| 1:2* | 4437 | 10061 |
| 1:3* | 691 | 735 |
| Assembled genome size (bp) | 783,486,840 | 673,403,748 |
| Gene number | 45,002 | 36,709 |

*These means there are 1/2/3 genes in *C. parvula* or *C. kokanica* have synteny relation with one gene in *C. littledalei.*


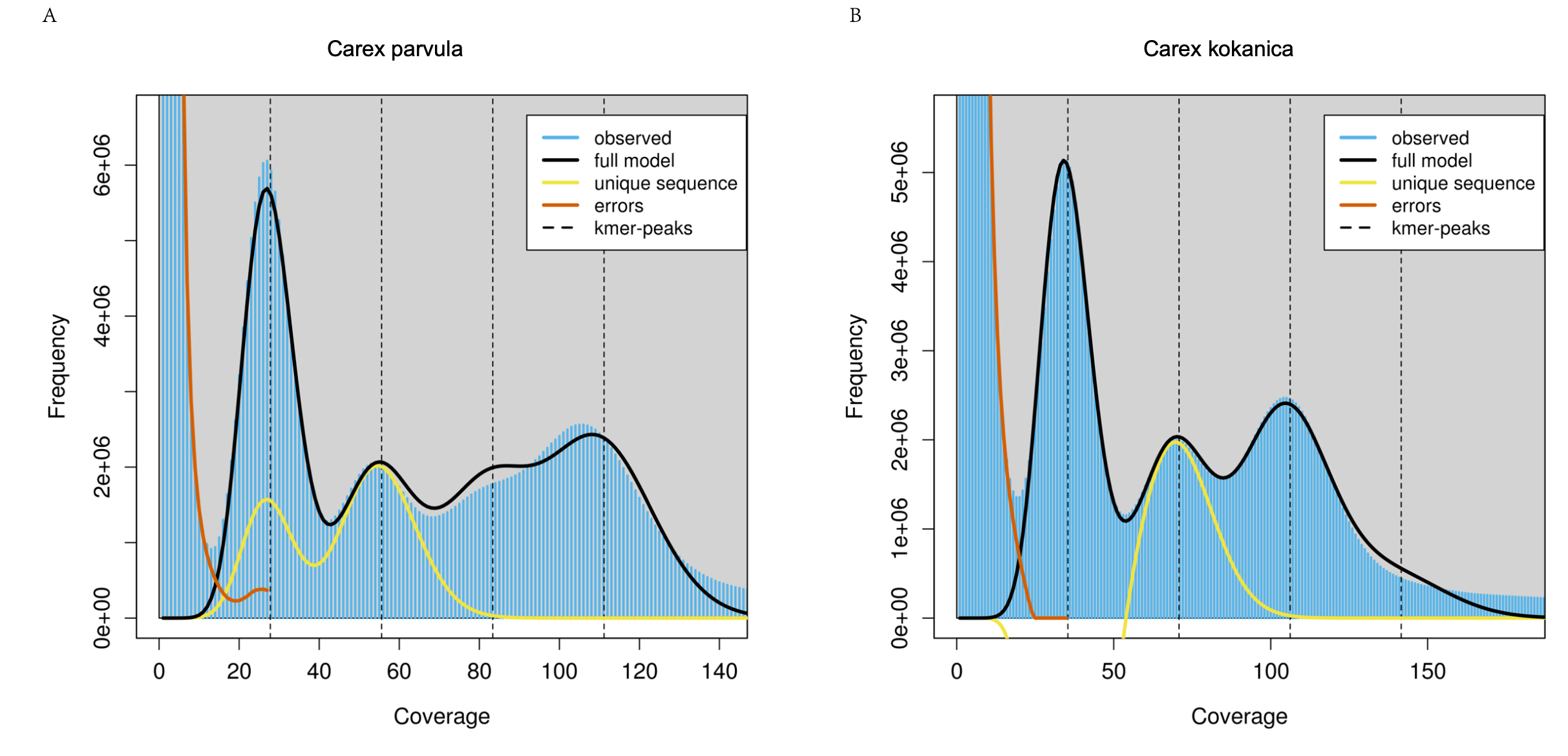


**Supplemental Figure S1.** **The K-mer distribution of Illumina paired-end reads. *Carex parvula* (A) and *Carex kokanica* (B).**


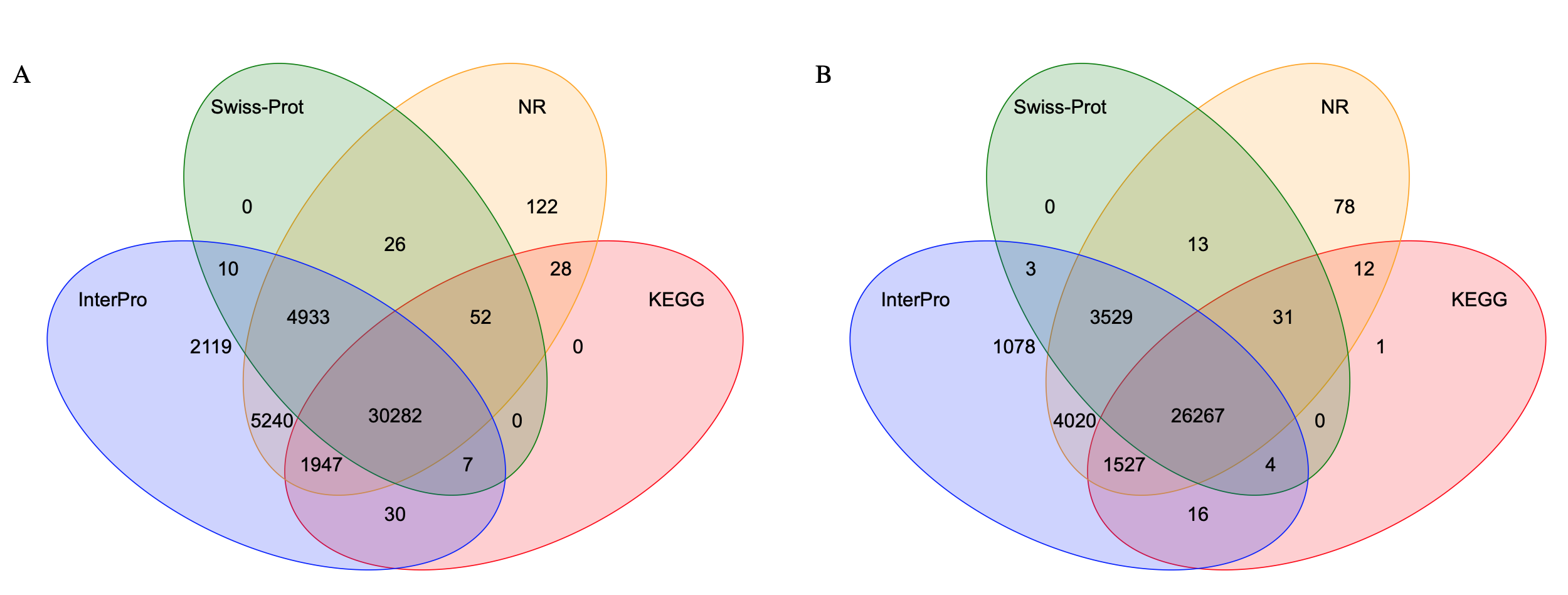
 **Supplemental Figure S2.** **The statistics of gene function annotation. *Carex parvula* (A) and *Carex kokanica* (B).**


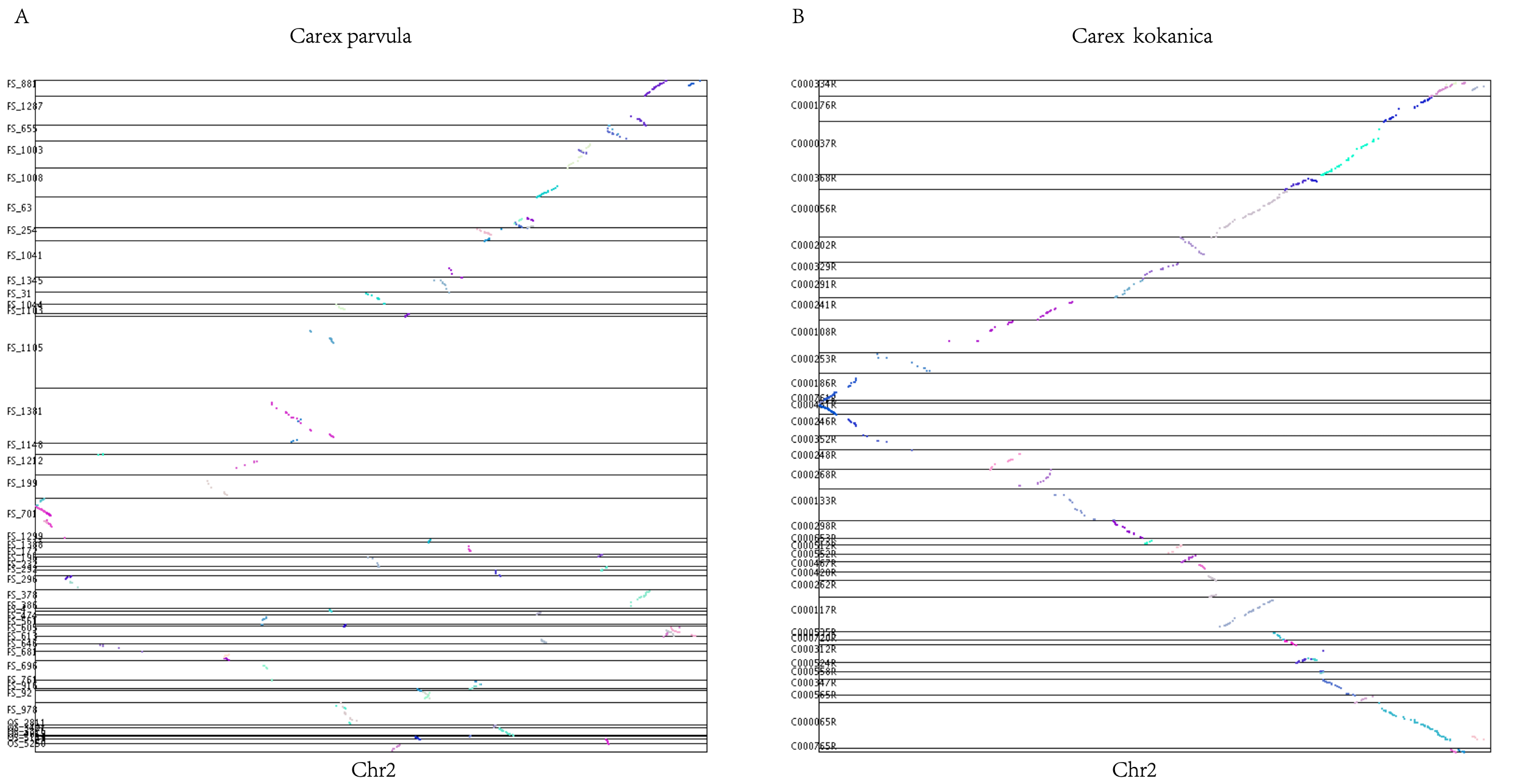


**Supplemental Figure S3. Comparison between contigs of *Carex parvula*(A), *Carex kokanica*(B) and *Carex littledalei* Chromosome 2.**

**
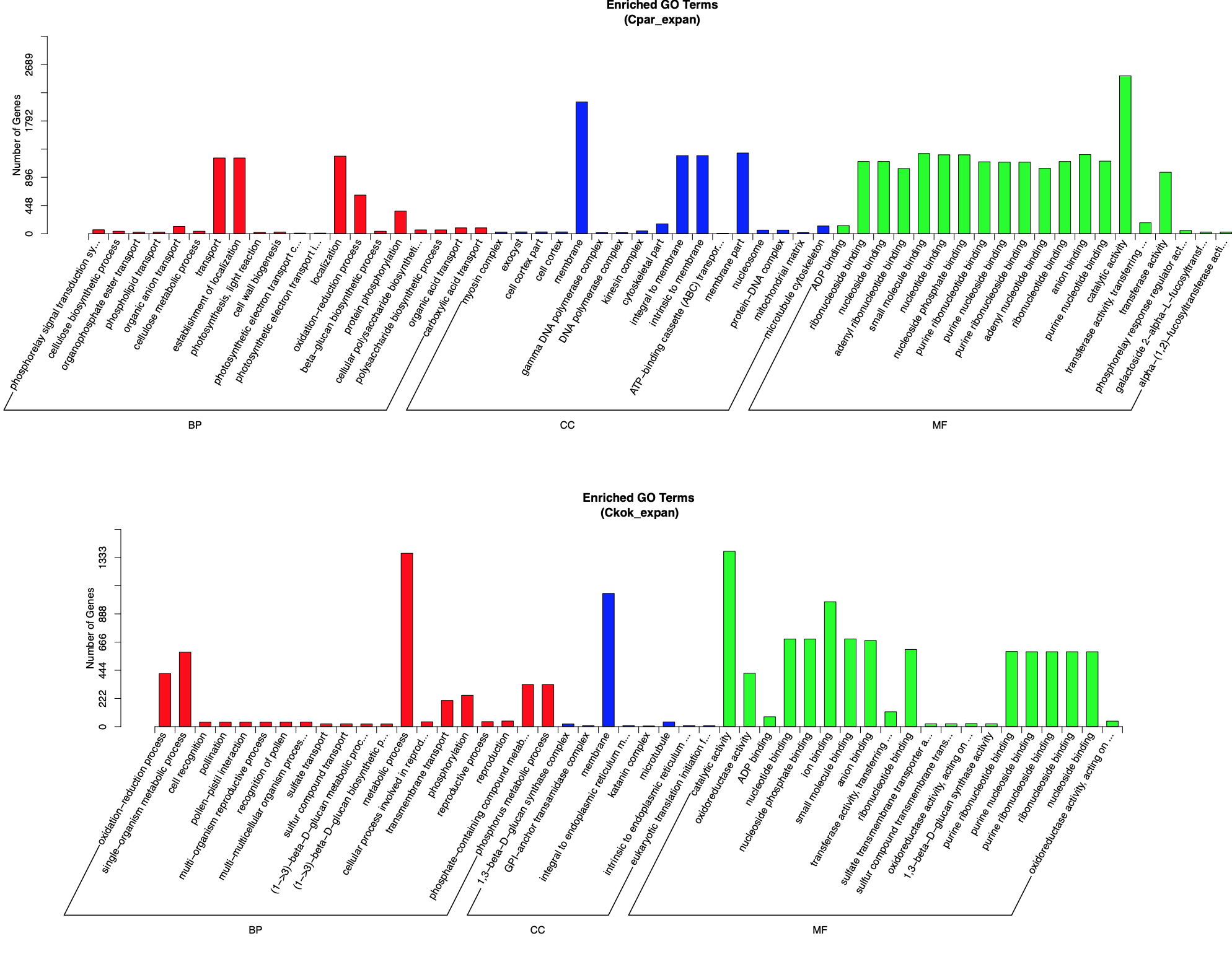
**

**Supplemental Figure S4. Enriched GO terms of the expanded genes of *Carex parvula* (Cpar) and *Carex kokanica* (Ckok).**
